# “*Can A Ballerina Eat Ice Cream?”*: A Mixed-Method Study on Eating Attitudes and Body Image in Female Ballet Dancers

**DOI:** 10.3389/fnut.2021.665654

**Published:** 2022-01-06

**Authors:** Heloisa C. Santo André, Ana Jessica Pinto, Bruna Caruso Mazzolani, Fabiana Infante Smaira, Mariana Dimitrov Ulian, Bruno Gualano, Fabiana Braga Benatti

**Affiliations:** ^1^School of Applied Science (FCA), State University of Campinas, Limeira, Brazil; ^2^Applied Physiology and Nutrition Research Group, Rheumatology Division, Faculdade de Medicina FMUSP, School of Physical Education and Sport, Universidade de São Paulo, São Paulo, Brazil; ^3^Department of Nutrition, School of Public Health, University of São Paulo, São Paulo, Brazil

**Keywords:** classical ballet, eating disoders, disordered eating, body image, restrictive dieting

## Abstract

**Aim:** We aimed to explore how a group of classical ballet dancers perceived their eating attitudes and their bodies, with special attention to the potential presence of eating disorders (EDs) symptoms and body image (dis)satisfaction.

**Methods:** A cross-sectional, mixed-method study was conducted on fourteen trained classical ballet dancers (18–30 years old). Their experiences, perceptions, and feelings regarding eating attitudes and body image concerning classical ballet were acquired through qualitative focus groups. The presence of EDs symptoms and perception and (dis)satisfaction with body image was analyzed quantitatively through self-report questionnaires.

**Results:** Participants reported concerning eating attitudes during the focus groups, such as the regular practice of several restrictive popular diets, constant restriction of foods considered “heavy” or “fatty,” meal skipping and ignoring signs of hunger, presence of overeating episodes due to stress and anxiety, feeling guilty about breaking their usual diet, classifying foods as “good” and “bad” or “lean” and “fat,” and excluding some of those foods from their usual diets. These reports were partially reflected in the questionnaires, with 50% of the ballerinas showing bulimic symptoms indicative of an unusual eating pattern (only two of them with a significant risk index), 7.1% showing symptoms of moderate binge eating, and 14.3% symptoms of EDs in general. Additionally, when considering their bodies in the context of everyday life, participants were satisfied; however, in the “classical ballet” context, they reported feeling dissatisfied with their shape. These findings were in line with results from the Stunkard's Scale, which revealed that 50% of the sample was dissatisfied with their current body shape and 57.1% indicated that their desired body shape was a leaner figure than one they considered healthy.

**Conclusions:** The constant practice of restrictive diets and other weight-loss strategies to achieve a leaner body were associated with symptoms of EDs and body dissatisfaction in this sample. Importantly, the questionnaires used seemed to underestimate the presence of a disordered eating pattern reported by the participants during focus groups. These data could help to inform psychological and nutritional strategies aimed at improving performance, physical and psychological well-being, and quality of life of ballet dancers.

## Introduction

Eating disorders (EDs) are psychiatric disorders with multifactorial origins, which can cause major biopsychological damage and increased mortality (1). EDs affect mainly adolescents and young adults, particularly females (2). Moreover, a few subgroups of the population seem to be at an even higher risk of developing EDs, for instance, the prevalence of EDs in athletes is higher than in the general population and greater among female athletes (3, 4). Furthermore, the risk of developing EDs is also higher in sports modalities that have an aesthetic component or categorize athletes according to weight (5).

EDs in athletes may have major health and performance consequences, namely, premature muscle fatigue, impaired thermoregulation, impaired oxygen and nutrient transport, reduced aerobic capacity, bone loss, increased susceptibility to infections, anemia, gastrointestinal disorders, and dehydration (6), in addition to increased susceptibility to depression, anxiety, and low self-esteem (7).

Classical ballet is a physically demanding sport characterized as an intermittent exercise, which demands energy from different metabolic pathways (8). Moreover, it is an artistic expression in which the demand for a lean body is broadly accepted as necessary for dancers to succeed (9), considering the high demand in terms of uniform, such as leotard and tights, and the high number of movements which require lifting (10). This cultural “requirement” might potentially explain a prevalence rate of 16.4% of EDs in classical ballet dancers and a 78% higher risk of developing these disorders when compared with non-dancers (11).

Researchers have attributed the higher risk of developing EDs in classical ballet dancers to their characteristics such as perfectionism (12), low self-esteem (13), body image dissatisfaction, (13) and even genetic predisposition (12). Moreover, when compared with dancers from other genres, classical ballet dancers show a higher percentage of body mass index lower than 17.5 kg/m^2^, combined with more pressure from choreographers to achieve a lean body (10, 14).

Previous qualitative research has also investigated specific risk factors which may make dancers more susceptible to the development of EDs. Thinness-Related Learning (TRL), which is the degree to which an individual is exposed to learning about thinness in a dance class, such as comments from teachers and peers about the benefits of dieting, social comparisons between peers and observational learning of dieting and restriction through a teacher or peer modeling, may play a role (15). Indeed, food restriction and the pressure to be thin are characteristics commonly reported by dancers in the classical ballet environment (16).

Despite these valuable findings, no study has investigated specific eating attitudes associated with EDs and body image in classical ballet dancers by combining both quantitative and qualitative approaches, which may allow for a more in-depth investigation into this relationship and help understand associated feelings and perceptions. Based on previous literature, we expected to better understand how the participants' rehearsal and championship routines, the relationship with their coaches and peers, and their career goals related to their eating attitudes, the relationship with their bodies, and the presence of EDs symptoms. This approach may also lead to new areas of investigation, in this case, new behaviors and attitudes potentially associated with EDs in this population. Thus, this study aimed to explore experiences, perceptions, and feelings regarding eating attitudes associated with EDs symptoms and body image (dis)satisfaction in classical ballet dancers using a mixed-method approach.

## Materials and Methods

### Study Design

A cross-sectional, mixed-method study was conducted. Experiences, perceptions, and feelings regarding eating attitudes and body image in relation to the classical ballet were qualitatively explored through focus groups. Additionally, the presence of EDs symptoms and perception and (dis)satisfaction of body image was analyzed quantitatively through self-report questionnaires.

#### Participants

Four Ballet schools in the city of Campinas, São Paulo - Brazil, were contacted. Two schools declined to participate in the study. All dancers from the two schools who agreed to participate took part in the study.

The convenience sample consisted of 14 female ballet dancers, aged between 18 and 30 years (22 ± 4 years) with a BMI between 17.8 and 22.0 kg/m^2^ (20.6 ± 2.1 kg/m^2^) who regularly trained in ballet schools (more than 4 times a week). Participants had been practicing ballet for an average of 14 ± 7 years and 8 of the 14 participants were professional ballet dancers at the time of the study. The other participants of the study (6) were not professional dancers, although they still took regular ballet classes and competed in some local ballet competitions.

All participants signed the Informed Consent Form (CAAE: 95432818.5.0000.8142), approved by the Research Ethics Committee of the State University of Campinas (UNICAMP) before entering the study.

### Qualitative Methods

Aiming to explore the dancer's experiences, perceptions, and feelings regarding their eating attitudes and body image in relation to classical ballet, three focus groups were conducted (17), each composed of 3, 5, and 6 dancers. Focus groups were led by a moderator, who was an experienced researcher on qualitative methods, to provide a judgment-free atmosphere in which participants could feel comfortable expressing their opinions. The moderator used pre-defined, open-ended questions (Supplementary File 1), which aimed to guide the conversation and ensure that all necessary topics were covered. The questions were first created by two researchers who discussed the objectives of the study and raised aspects they considered relevant to be addressed. Thereafter, questions were either approved or vetted by three other researchers, two of whom were experts in EDs and one in sports science, leading to the final questions used in the study.

During focus groups, we asked participants to report experiences and thoughts about their rehearsal routine; features of their usual diets; changes in their usual diets when close to championships, presentations, or vacations; whether they compared their diets with non-dancers' diets; whether other people make comments about their eating patterns and how they felt regarding these opinions. We also asked if they had previously followed restricted diets and why; which foods they “cannot” eat during their usual diets and how they dealt with any potential desires to eat these foods; what they eat after championships or presentations; what was a pleasurable meal for them and how they felt when they ate it. Moreover, we raised questions about what they felt regarding their bodies in different contexts (i.e., in the context of classical ballet and in everyday life); whether they received comments from others about their bodies, and how they dealt with those. Finally, we asked questions about their careers, including their career goals, what they would do to achieve them, and what their diets would look like if they stopped dancing. Focus groups were not used to assess the frequency or the extent of disordered eating attitudes and behaviors. They took place at the dance schools, in a private room with no contact with the school's coaches or staff, lasted between 30 and 45 min, were recorded, and later transcribed verbatim to enable qualitative analysis.

### Quantitative Methods

The presence of EDs symptoms was assessed quantitatively *via* three self-administered questionnaires, all translated into Portuguese and previously validated for the Brazilian population (18–21).

To assess eating attitudes and behaviors, we used the Eating Attitude Test (EAT-26) (22). This is a self-administered questionnaire composed of 26 questions across 3 categories: (1) Dieting: Reflects a pathological refusal to eat high-calorie foods and intense preoccupation with body image; (2) Bulimia: Refers to episodes of excessive food intake, followed by vomiting and other behaviors to prevent weight gain; (3) Food Preoccupation: Refers to self-control over food and recognized social forces in the environment that stimulate food intake. Each question has 6 answer options (always = 3, often = 2, sometimes = 1, few times = 0, almost never = 0, never = 0), and total score can range from 0 to 78. Scores ≥ 21 correspond to abnormal eating behavior and increased risk of ED.

The Bulimic Investigatory Test Edinburgh (BITE) aims to identify individuals with ED, specifically bulimia nervosa, and to evaluate control over eating behaviors (23). It consists of 33 questions divided into 2 parts, referring to symptoms and their severity. All questions relating to symptoms (all questions apart from 6, 7, and 27) are answered “yes” or “no,” with corresponding scores of 1 or 0 points. Questions 6, 7, and 27 relate to the severity of the symptoms and are scored on scales ranging from 1 to 5 (Q.6) or 1 to 7 (Q.7 and 27). On the symptom scale, a score > 10 is representative of atypical eating behaviors, while scores > 20 indicate compulsive, restrictive, or purgative behaviors. On the severity scale, a score ≥ 5 indicates that symptoms may be clinically significant, while scores ≥ 10 indicate high severity.

To assess binge eating and the severity of symptoms, we used the Binge Eating Scale (BES) (24). BES is composed of 16 questions, each with 3 or 4 answer options scoring from 0 to 3 (0 = absence of symptom and 3 = severe symptom). The overall score can range from 0 to 46. Scores ≤ 17 indicate the absence of binge-eating behaviors; scores between 18 and 26 indicate moderate binge-eating behaviors and scores ≥ 27 indicate severe binge-eating behaviors.

Perception of body image was assessed using the Stunkard's Scale (25), which consists of nine female figures, numbered 1–9, ranging from a very thin figure (# 1) to a very obese figure (# 9). Participants should choose three figures, one they believe best represents their current body, one they believe represents a desirable body, and one they consider to be a healthy body. Scores were calculated by subtracting the current body choice by the desirable body and healthy body choice. Negative scores represent body dissatisfaction.

### Data Analysis

Quantitative data are presented as mean ± standard deviation (range), unless stated otherwise. The qualitative data (focus groups) analysis approach involved classical and exploratory content analysis (26). The qualitative data analysis was conducted with the software MAXQDA. This is a world-leading software package for qualitative and mixed methods research. Its interface offers an experience very similar to that provided by Windows text editor, creating an easy-to-use, and user-friendly experience. The researchers were trained before its use by an experienced qualitative researcher. Classical content analysis used themes derived from the EDs literature and questionnaires (used in the quantitative analysis) and were defined *a priori* (i.e., presence of symptoms consistent with EDs and body image satisfaction). Further themes arose from the data by exploratory content analysis, totaling six themes which are described in Table 1. Initially, a first reading of the material was performed and the corpora of the work was defined. A codebook was developed for coding these themes, which included for each theme: short and detailed descriptions; inclusion and exclusion criteria; typical and atypical quotes, and an exemplar classified as “close but no” (26, 28). Two researchers discussed the codebook and applied it independently to the corpora, using phrases as the unit of analysis. The “cutting and sorting” approach, a process that identifies important quotes and expressions and then arranges them according to similarities, was used to identify the themes (26). Kappa coefficients for inter-rater reliability were calculated using the GraphPad QuickCalcs, with values between 0.61 and 0.80 defined as “good” agreement and ≥0.80 as “very good” agreement (Table 1) (27). Speeches from participants are identified as “P.”

**Table 1:** Codes and kappa coefficients from focus groups.

**Theme**	**Kappa**
Usual eating	0.750 ± 0.079
Unusual eating	0.929 ± 0.025
Eating attitudes	0.894 ± 0.037
Body image	1.000 ± 0.000
External perceptions	1.000 ± 0.000
Reharsals and career goals	0.876 ± 0.046

*Kappa results are interpreted as follows: 0.61 to 0.80 as “good agreement,” and 0.81 to 1.00 as “very good agreement” (27)*.

## Results

### Participants Characteristics

According to the EAT-26 questionnaire (Table 2), twelve dancers (85.7%) showed no restrictive-eating behaviors or symptoms consistent with the presence of EDs, while two dancers (14.3%)—both professionals—had scores higher than 21 points (22 and 36), which suggests the presence of EDs symptoms. Regarding BES, only one dancer (7.1%)—also a professional—presented symptoms of moderate binge eating (25 points), while thirteen dancers (92.8%) did not present binge-eating symptoms (Table 2).

**Table 2:** Presence of ED symptoms.

**Outcome**	**Mean ± SD**	**Range**
**Presence of eating disorders symptoms**		
BES	7.9 ± 6.7	1–25
BITE (symptoms)	8.4 ± 4.6	2–15
BITE (severity)	2.1 ± 1.7	0–5
EAT	9.1 ± 9.7	1–36
**Body image perception and dissatisfaction**		
Body satisfaction score	−0.6 ± 0.6	−2–0
Stunkard's scale (current)	3.2 ± 0.9	2–5
Stunkard's scale (healthy)	3.3 ± 0.6	2–4
Stunkard's scale (desirable)	2.7 ± 0.6	2–4

*BES, Binge Eating Scale; BITE, Bulimic Investigatory Test; EAT, Eating Attitude Test. Data presented as mean ± standard deviation*.

From the symptom scale of the BITE questionnaire, 7 dancers (50.0%) showed bulimic symptoms, obtaining a score between 10 and 19 (medium score, suggesting unusual eating patterns), 6 of them being professionals. The other 7 dancers (50.0%) of the sample presented low scores. Moreover, on the severity scale, only two participants (14.3%), also professional ones, presented clinically significant scores whereas the remaining participants presented low scores (Table 2).

**Figure 1:**
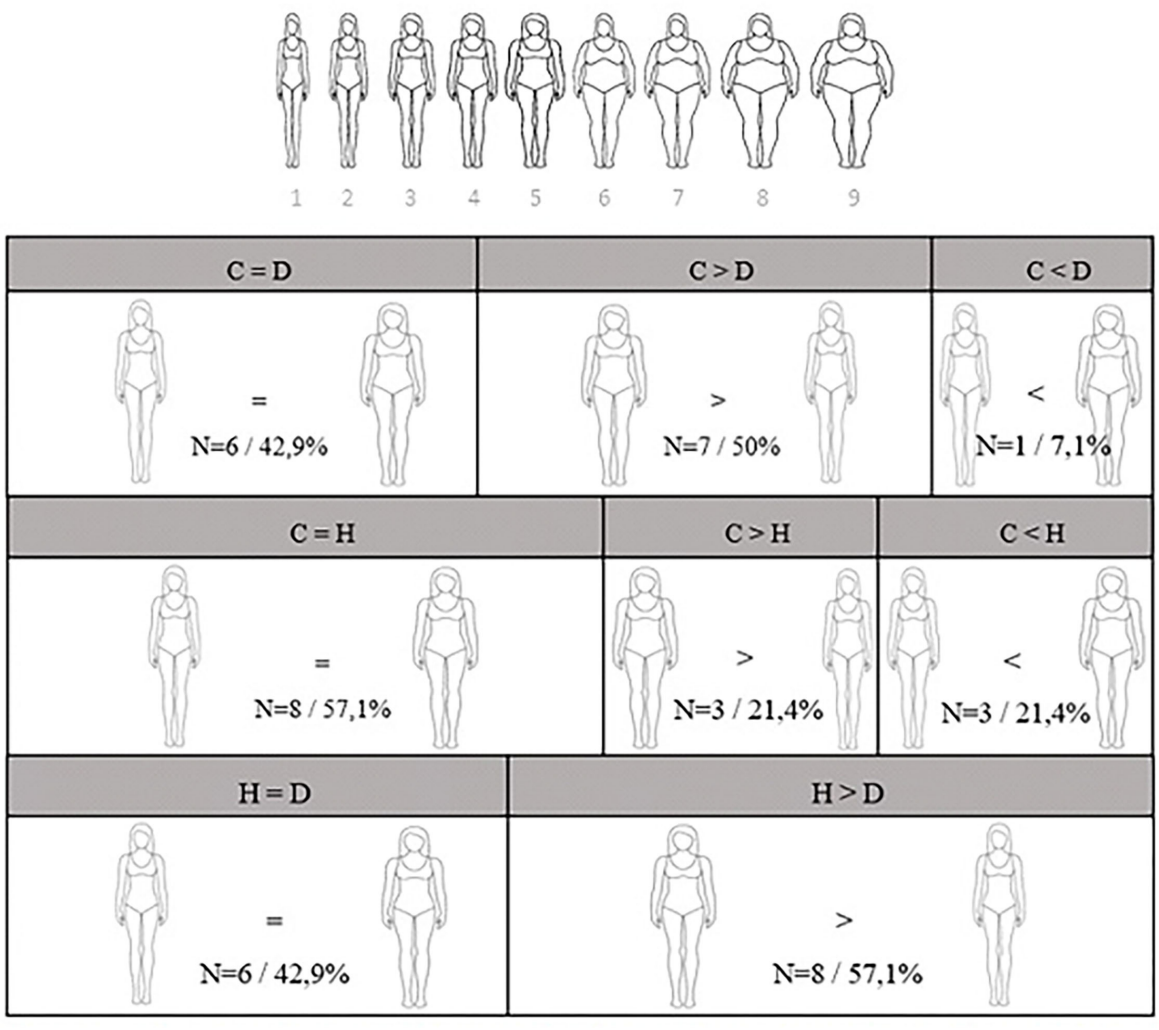
Frequency of reporting in different Stunkard's Scale categories. C, current body shape; D, desirable body shape; H, Healthy body shape. Images adapted from Stunkard's scale.

Regarding body image perception, 7 dancers (50.0%), 4 of them being professionals, wanted to have a thinner body than their perception of their current body shape, while 6 of them (42.9%), 4 of them being professionals, were satisfied with their current body shape according to the Stunkard's scale (Figure 1). More than half of the dancers (57.1%)−2 professionals—indicated that their desirable body shape was a leaner figure than the one they considered healthy, suggesting that they did not exactly perceive their desirable body shape to be healthy. Eight dancers (57.1%)−4 professionals—considered their current body to be healthy, while three of them (21.4%)−1 professional—believed that their current body shape was thinner than the one they considered to be healthy (Figure 1).

### Experiences, Perceptions, and Feelings Regarding Eating Attitudes and Body Image in Relation to Classical Ballet

#### Usual Eating

When asked about their usual eating pattern, dancers agreed on the importance of having a dietary routine for performing as a dancer: “*Here (in the ballet school) we have a tight schedule, so we must have a healthy dietary routine in order to have energy and feel willing to take the dance classes and rehearsals” (P3)*. They also emphasized the fact that they stick to a regular diet during the week, allowing for some exceptions on the weekend: “*I control myself during the week so I can eat an ice cream on the weekend” (P5)* or “*From Monday to Saturday I eat healthy foods, on Sundays I usually falter and have a milk shake, go to the mall, cinema, popcorn, all these things“(P7)*. Participants talked about skipping meals, especially dinner, or cutting out specific foods such as sodas, fried foods, sweets, or even rice during weekdays either as a dietary strategy or due to rehearsal schedule issues: “*On weekdays I eat ‘right’: I don't drink soda, I eat lots of eggs, I don't eat dinner, these things” (P5), “When I have rehearsals at night, I don't have dinner, because when I get home late, I don't get hungry” (P11)*. Another issue brought up by the dancers was that eating fatty foods or foods they considered as ”heavy“ lead to increased fatigue and decreased performance during classes and rehearsals: ”*if you eat junk food on a day that you have rehearsals, like Saturday, it ruins it” (P4)*.

#### Eating Attitudes

Importantly, dancers expressed concern during periods when they step out of their usual diet, leading to an “all or nothing” eating behavior [“*On weekends, I throw everything up in the air” (P1); “When I start a restrictive diet, I stay on focus until the end of it, but I know that, on the next day, I'll eat whatever I want” (P8)*]. Episodes of overeating were also brought up by some ballet dancers when it came to eating what they considered to be “pleasurable foods”: “*One day I picked up a Nutella jar and I ate it and cried later” (P1) “When I eat sweets, I feel heavy and I feel a little remorse” (P12)*. The dancers also mentioned strategies for not deviating from their diets and eating foods considered “bad” or “fatty”: “*I drink a lot of water. Are you hungry? Drink some water!” (P4);* or “*At night, if I already had dinner, but I'm still hungry, I don't eat, I usually sleep” (P1)*.

#### Unusual Eating

The role of dieting in a dancer's life was also discussed. All of them said that at some point, they tried to follow popular restrictive diets, such as “Detox,” “Low-carb,” and “Keto-diet.” When asked about possible motives which had led them to undergo these types of diet, different explanations came up, such as “*Desire to see immediate results” (P5)*; “*I did it due to lack of maturity” (P6)*; “*To improve my performance! My body had to work harder and recover faster” (P2);* or “*Aesthetics, the dancer always has to be thin” (P7)*; or even the influence of social media [“*I always saw the nutritionists on Instagram saying that ‘low-carb’ is good for this and that” (P7)*].

When it came to pre-championships or pre-dance festivals, dancers agreed that they usually change their eating pattern during times of increased rehearsal hours, anxiety, and when feeling fat while dressing up: “*When it gets closer to a competition or dance festivals, we feel anxious and we eat a little more or a little less, it depends, but the emotional part counts a lot. Also, when we have a greater load of rehearsals, we end up skipping meals, otherwise we feel heavy” (P3); “If I want to eat some chocolate, I think ‘I'm too close to the presentation, I won't eat it”’ (P7)*.

Dancers also debated types of food they eat after championships and dance festivals [“*Almost every time we compete, we have a burger or a pizza afterwards” (P9)*] and motives underlying these behaviors [“*It's kind of a reward, to celebrate” (P12)*].

When asked if they would change something in their usual diets if they stopped dancing, the dancers said they would not change much, just adapt to a new routine: “*Maybe a less restricted and worrying diet but always looking for a balance” (P8)*.

#### External Perceptions

The relationship of participants with external opinions such as family, friends and colleagues regarding their eating habits was also discussed: “*My dad is always watchful with the things that I eat, and he constantly says things like ‘oh, you already ate that, you won't eat anymore’, and I know he does that because of the ballet” (P13)*; “*When I'm with someone who's not a dancer and they see me eating an ice cream for example (...) they often say: ‘Wow, can a ballerina eat ice cream?” (P7); “I am a vegetarian and I usually eat in healthy restaurants, so my friends constantly say that, because I am a ballerina, I am fussy about food and that I am ‘fitness”’ (P14)*. When dancers were asked about the consequences of these external opinions on their attitudes, the answers were mixed. Many said they do not care; some said they do not care anymore, but that at some point in their lives these comments caused some discomfort; and others said it bothers them: “*When I was younger, it bothered me a lot, I think it passed over time, about a year and a half ago I stopped caring about those comments” (P8)*; or “*From the moment a person looks at me and says thinks like ‘Are you really going eat this?’, I eat the whole thing thinking that I shouldn't eat it” (P1)*.

#### Body Image

Perceptions, attitudes, and concerns of ballet dancers toward their bodies were discussed during focus groups. Participants' perceptions toward their bodies varied when they put it into context: “*When I look in the mirror like a normal person, I feel good, but if I look in the mirror as a ballerina, I want to die” (P1)*; “*When it comes to ballet practice, I get more concerned about my body, since there is always someone correcting me, telling me to close my chest, or shrink my belly, so I get more uncomfortable” (P11)*.

Dancers also mentioned situations where they heard external opinions about their bodies: “*Once I heard from one (teacher): you are not fat, but you have wide knuckles” (P9); “Actually, I always heard people say that I am too skinny, and I suffered a lot with this” (P10); “There was a teacher one time that told me to lose butt fat” (P13)*. Interestingly, however, most of them said they did not care about these opinions regarding their bodies: “*If I cared I would go crazy” (P13)*.

#### Rehearsals and Career Goals

In addition, the participants agreed that there is a predefined stereotype assigned to the classical ballet dancers, and that it had a large influence on whether they wanted to become a professional: “*I don't have the ideal ballet dancer body type, I'm lucky that I wasn't born with that dream (...), I see myself more on the side of the contemporary dance, where my body fits better” (P2)*. / “*To be professional, there is a pattern that ‘they’ want, and to say that I want it to be a professional, it's kind of a utopia, I would like that, but there is this stereotype factor” (P12)*.

## Discussion

The aim of this study was to explore experiences, perceptions, and feelings regarding eating attitudes associated with EDs symptoms and body image (dis)satisfaction in a cohort of ballet dancers, using a mixed-method approach.

During focus groups, eating attitudes related to the regular practice of several restrictive popular diets, constant restriction of foods considered “heavy” or “fatty,” meal skipping and ignoring signs of hunger were identified in our participants. Moreover, overeating episodes due to stress and anxiety, feeling guilty about breaking their usual diet (using expressions like “*I threw everything up in the air*” or “*I flinched*”), classifying foods as “good” and “bad” or “lean” and “fat,” and excluding some of those foods from their usual diets were also reported by these ballet dancers. These reports of disordered eating were partially reflected in the questionnaires, with 50% of the ballerinas showing bulimic symptoms indicative of an unusual eating pattern (only two of them with a significant risk index), 7.1% showing symptoms of moderate binge eating, and 14.3% symptoms of EDs in general.

A strong association between the practice of weight-loss diets and the development of EDs has been extensively shown in the literature (29). A study that tracked 1,000 teenage girls for 1 year showed that the relative risk of a dieter developing EDs is eight times higher than a non-dieter, thus concluding that dieting is a strong risk factor for the development of EDs (30). Since many of the ballet dancers in the study reported the habit of regular dieting in addition to disordered eating symptoms on the questionnaires, it is more than possible that these symptoms may develop into clinical EDs. Ballet dancers and coaches should be made aware of these dangerous practices to reduce the risk of these athletes developing serious EDs, which may impact their overall health.

The participants also talked about applying certain strategies, such as drinking water or going to sleep when feeling hungry, to prevent themselves from eating “junk food” or foods that did not form part of their usual diets, particularly near competitions and dance festivals. This may explain the higher symptoms score on the BITE questionnaire compared to the BES and the EAT-26, since the symptoms part of BITE is composed of “yes” or “no” questions, while BES and EAT-26 are related to the frequency of behaviors. Thus, although only 7–15% of the sample reported a high frequency of these behaviors, they were present in 50% of the sample, probably around competitions.

It is noteworthy that the questionnaires used in the study seemed to underestimate the presence of a disordered eating pattern in the participants, with relatively low scores, particularly in the EAT-26 and BES. This apparent discrepancy between qualitative and quantitative findings is not necessarily uncommon and has been reported in the previous studies (31). The questionnaires aimed at tracing ED symptoms might have also introduced an artificial element to the way the participants assessed their “dysfunctional” eating patterns, leading them to soften their understanding of dysfunctional eating behavior. The qualitative tools used in this study aimed to explore their experiences, perceptions, and feelings regarding their eating attitudes and body image. If we consider these topics as prompts to talk about their behaviors, it makes sense that the “dysfunctional eating” compartment was assessed in the participants with this approach. It is possible to assume that the mixing of study designs (i.e., qualitative and quantitative) used herein enabled us to find “blind spots” which would not have been assessed using the quantitative approach alone. Moreover, it may be that these questionnaires use more rigorous definitions of disordered eating behaviors, which may underestimate their incidence in the sample. Therefore, the use of qualitative approaches might be useful and complementary to questionnaires to assess the risk of developing EDs in this population.

These eating attitudes were potentially driven by the dancers' perception of their body image, as 50% of the ballet dancers showed some dissatisfaction with their current body shape. Although we cannot specifically say we found evidence of body dysmorphia, the fact that more than half of the participants (eight of them) considered a desirable body to be one thinner than a body they considered to be healthy is somewhat concerning. Previous research has demonstrated that dancers express more concerns about their physical appearance and body image, which, according to them, have an impact on how they control their dietary intake and eating habits continuously, to lose or maintain weight (32). Accordingly, Kulshreshtha et al. (33) showed a positive correlation between body dissatisfaction and disordered eating attitudes, concluding that dancers who are more concerned with body shape size were five times more likely to report disordered eating attitudes than their peers.

These data support the so-called “dancer's stereotype,” mentioned by the participants themselves in the focus groups, which is characterized by a slim and light body (34). It is noteworthy that during focus groups, participants mentioned differences in body satisfaction when dancers compare how they see their bodies in and out of the classical ballet context. They pointed out that when they analyze their bodies in a more regular context (i.e., everyday life), they consider themselves satisfied; however, when analyzing themselves in the classical ballet context, they would prefer their bodies to be thinner, which may explain the fact that 50% of the sample showed body image dissatisfaction.

Literature shows that when compared with non-dancers, dancers have a higher risk of developing EDs, disordered eating attitudes, more dieting behaviors, and more binge-eating episodes (32, 33). Moreover, compared to other dance genres (contemporary, flamenco, and Spanish dance), ballet dancers are more likely to appoint their coaches as a key factor in their body dissatisfaction, and to report more pressure from choreographers (14, 35), an issue that has also been brought up by the dancers in the focus groups, when they talked about how common it was for coaches to talk about their bodies.

It is noteworthy that the majority of participants reporting the highest scores in the questionnaires were professionals, indicating that they are at the greatest risk of developing ED than the students. Body image is known to play a significant role in the process of “socialization” among classical ballet dancers (36). Alexias and Dimitropoulou (36) argue that “professional ballet dancers form an almost ascetic, abstinent attitude toward their bodies in order to have it work at the limits of its biological basis (overcoming even pain and serious injuries) and offer them profits (in a wider sense including financial remuneration as well as prestige and professional development and distinction)”. This gives support to the association between body dissatisfaction and a higher risk of developing EDs, particularly in sports modalities with a strong aesthetic component such as classical ballet (16).

A study conducted in 2013, which evaluated 237 girls in Spain, showed that there is an indirect effect of using social media with body dissatisfaction, as current media can generate greater competitiveness and comparisons between people, which may lead to a negative body image (37). The influence of media was also raised in the focus groups and specified as a reason to begin dieting, especially “low-carb” diets. This factor exemplifies the dancers' lack of knowledge about nutrition, which is often exploited in social media by personalities who constantly promote “miraculous diets” with impressive results that lack scientific underpinning. Dancers, who often lack adequate professional and nutritional support, end up believing bogus claims and following radical suggestions. In this regard, it is worth mentioning that only a few participants underwent nutritional monitoring, suggesting a low participation of skilled professionals in classical ballet. Nutritional support could be extremely important in the prevention and treatment of EDs symptoms in dancers, as well as helping them to improve performance and quality of life.

The strengths of this study include the recruitment of some experienced and professional ballet dancers who have been engaged in this sport for a long time, allowing determination of chronic exposure to the “classical ballet environment” on body image and EDs. Moreover, the qualitative analysis allowed an in-depth investigation of behaviors related to body satisfaction and EDs. The limitations include the relatively small, convenience sample enrolled in the study, as participants were selected from two schools only, limiting the generalizability of findings. It is noteworthy, however, that although a sample size of 14 may be regarded as small for studies essentially composed by quantitative methods, it was the qualitative component that guided our findings, whereas the quantitative component was used to characterize the sample and to complement the qualitative data. Importantly, our data showed that the information that emerged from qualitative methods reached saturation, as themes surfaced in different focus groups were quite similar, indicating that the sample size was adequate for the main aim of the study. Additionally, although we made a strong effort to provide a welcoming and judgment-free environment, the ballet dancers might not have been completely open in the completion of questionnaires and during focus groups. It is worth mentioning, however, that focus groups were characterized by the expressive participation of the participants and the discussion of a wide variety of ideas, suggesting that participants did feel comfortable expressing their opinions. Finally, it is possible that other factors out of the scope of the present study, such as menstrual status, may have influenced the results. Future research should address these factors.

## Implications for Research and Practice

In conclusion, the constant practice of restrictive diets and other weight-loss strategies to achieve a body shape leaner than the one they considered healthy were associated with symptoms of EDs and body dissatisfaction in a sample of female ballet dancers. Of importance, body dissatisfaction was shown to be associated with the sport itself, as the dancers did not report any sign of dissatisfaction with their bodies in the context of “normal societal standards.” Thus, health professionals should consider assessing a dancer's body image (dis)satisfaction both in the general context and in the context of classical ballet, and exercise caution when recommending restrictive diets to achieve a leaner body, as they may lead dancers to the development of EDs. Moreover, the questionnaires used in the study seemed to underestimate the presence of a disordered eating pattern reported during focus groups by the participants. Therefore, health professionals should acknowledge that classical ballet dancers, particularly the professional ones, may present disordered eating behaviors before the development of EDs. These data are of clinical relevance and could be used to inform psychological and nutritional strategies to improve the performance, well-being, and quality of life of these dancers.

## Data Availability Statement

The raw data supporting the conclusions of this article will be made available by the authors, without undue reservation.

## Ethics Statement

The studies involving human participants were reviewed and approved by Research Ethics Committee of the State University of Campinas (UNICAMP). The patients/participants provided their written informed consent to participate in this study.

## Author Contributions

HS and FB conceived the study, analyzed and interpreted the data, and drafted the manuscript. AP, BM, FS, MU, and BG collected and analyzed the data. All authors revised and approved the final version of the manuscript and are, thus, accountable for its content.

## Funding

Funding for this study was provided by Conselho Nacional de Desenvolvimento Tecnológico, Coordenação de Aperfeiçoamento de Pessoal de Nível Superior, and Fundação de Amparo à Pesquisa do Estado de São Paulo (FAPESP; 2015/26937-4; 2017/13552-2). CNPq, CAPES, and FAPESP had no role in the study design, collection, analysis or interpretation of the data, writing the manuscript, and the decision to submit the paper for publication.

## Conflict of Interest

The authors declare that the research was conducted in the absence of any commercial or financial relationships that could be construed as a potential conflict of interest.

## Publisher's Note

All claims expressed in this article are solely those of the authors and do not necessarily represent those of their affiliated organizations, or those of the publisher, the editors and the reviewers. Any product that may be evaluated in this article, or claim that may be made by its manufacturer, is not guaranteed or endorsed by the publisher.

## References

[1] Philippi ST Cordás TA Salzano FT. General aspects of eating disorders. In: Manole, editor. Nutrition and Eating Behaviors: Evaluation and Treatment. 1st ed. Barueri: Manole (2011). p. 3–16.

[2] Hoek HW. Review of the worldwide epidemiology of eating disorders. Curr Opin Psychiatry. (2016) 29:336–9. 10.1097/YCO.0000000000000282 27608181

[3] Sundgot-Borgen J Torstveit MK. Prevalence of eating disorders in elite athletes is higher than in the general population. Clin J Sport Med. (2004) 14:25–32. 10.1097/00042752-200401000-00005 14712163

[4] Torstveit MK Rosenvinge JH Sundgot-Borgen J. Prevalence of eating disorders and the predictive power of risk models in female elite athletes: a controlled study. Scand J Med Sci Sport. (2008) 18:108–18. 10.1111/j.1600-0838.2007.00657.x 17490455

[5] Krentz EM Warschburger P. Sports-related correlates of disordered eating in aesthetic sports. Psychol Sport Exerc J. (2011) 2:375–82. 10.1016/j.psychsport.2011.03.004

[6] Beals KA. Effects of disordered eating on performance. In: Human Kinteics, editor. Disordered Eating Among Athletes: A Comprehensive Guide for Health Professionals. Champaign, IL: Human Kinetics (2004). p. 67–83.

[7] Swinbourne JM Touyz SW. The co-morbidity of eating disorders and anxiety disorders: a review. Eur Eat Disord Rev. (2007) 15:253–74. 10.1002/erv.784 17676696

[8] Rodrigues-Krause J Krause M Reischak-Oliveira Á. Cardiorespiratory considerations in dance: from classes to performances. J Dance Med Sci. (2015) 19:91–102. 10.12678/1089-313X.19.3.91 26349502

[9] Twitchett EA Koutedakis Y Wyon MA. Physiological fitness and professional classical ballet performance: a brief review. J Strength Cond Res. (2009) 23:2732–40. 10.1519/JSC.0b013e3181bc1749 19910802

[10] García-Dantas A Sánchez CDR Martín MS Navarro MLA Mas MB. Risk of Eating Disorders Among Different Dance Majorsat a Dance Conservatory. Vol 09 (2013). Available online at: http://institucional.us.es/apcs/php/index.php?option=com_content&view=article&id=237&Itemid=7 (accessed November 13, 2021).

[11] Arcelus J Witcomb GL Mitchell A. Prevalence of eating disorders amongst dancers: a systemic review and meta-analysis. Eur Eat Disord Rev. (2014) 22:92–101. 10.1002/erv.2271 24277724

[12] Thomas JJ Keel PK Heatherton TF. Disordered eating attitudes and behaviors in ballet students: examination of environmental and individual risk factors. Int J Eat Disord. (2005) 38:263–8. 10.1002/eat.20185 16211632

[13] Bettle N Bettle O Neumärker U. Body image and self-esteem in adolescent ballet dancers. Percept Mot Skills. (2001) 93:297–309. 10.2466/pms.2001.93.1.297 11693698

[14] Kalyva S Yannakoulia M Koutsouba M Venetsanou F. Disturbed eating attitudes, social physique anxiety, and perceived pressure for thin body in professional dancers. Res Danc Educ. (2021) 1:1–12. 10.1080/14647893.2021.1940124

[15] Annus A Smith GT. Learning experiences in dance class predict adult eating disturbance. Eur Eat Disord Rev. (2009) 17:50–60. 10.1002/erv.899 18729131

[16] Francisco R Alarcão M Narciso I. Aesthetic sports as high-risk contexts for eating disorders: young elite dancers and gymnasts perspectives. Span J Psychol. (2012) 15:265–74. 10.5209/rev_SJOP.2012.v15.n1.37333 22379716

[17] Krueger R Casey M. Overview of focus group. In: Sage Publication, editor. Focus Group: A Practical Guide for Applied Research. 4th ed. Thousand Oaks, CA: SAGE Publications (2009). p. 1–15.

[18] Cordas TA Hochgraf PB. The “BITE”: instrument to Bulimia Nervosa assessment: Portuguese version. J Bras Psiquiatr. (1993) 42:141–4.

[19] Duarte C Pinto-Gouveia J Ferreira C. Expanding binge eating assessment: validity and screening value of the Binge Eating Scale in women from the general population. Eat Behav. (2015) 18:41–7. 10.1016/j.eatbeh.2015.03.007 25880043

[20] Nunes MA Camey S Olinto MTA. The validity and 4-year test-retest reliability of the Brazilian version of the Eating Attitudes Test-26. Brazilian J Med Biol Res. (2005) 38:1655–62. 10.1590/S0100-879X2005001100013 16258635

[21] Scagliusi FB Alvarenga M Polacow VO. Concurrent and discriminant validity of the Stunkard's figure rating scale adapted into Portuguese. Appetite. (2006) 47:77–82. 10.1016/j.appet.2006.02.010 16750589

[22] Garner DM Bohr Y Garfinkel PE. The eating attitudes test: psychometric features and clinical correlates. Psychol Med. (1982) 12:871–8. 10.1017/S0033291700049163 6961471

[23] Henderson M Freeman CPL. A self-rating scale for bulimia: the “BITE.” *Br J Psychiatry*. (1987) 150:18–24. 10.1192/bjp.150.1.18 3651670

[24] Gormally J Black S Daston S Rardin D. The assessment of binge eating severity among obese persons. Addict Behav. (1982) 7:47–55. 10.1016/0306-4603(82)90024-7 7080884

[25] Stunkard AJ Sørensen T Schulsinger F. Use of the Danish Adoption Register for the study of obesity and thinness. Res Publ Assoc Res Nerv Ment Dis. (1983) 60:115–20. 6823524

[26] Bernard HR Wutich A Ryan GW. Content analysis. In: Sage Publications, editor. Analyzing Qualitative Data: Systematic Approaches. 2nd ed. Thousand Oaks, CA: SAGE Publications (2017). p. 243–68.

[27] Cohen J. A coefficient of agreement for nominal scales. Educ Psychol Meas. (1960) 20:37–46. 10.1177/001316446002000104

[28] MacQueen K McLellan E Kay KMB. Codebook development for team-based qualitative analysis. Cult Anthr Methods. (1998) 10:31–36. 10.1177/1525822X980100020301

[29] Loth KA Maclehose R Bucchianeri M. Predictors of dieting and disordered eating behaviors from adolescence to young adulthood. J Adolesc Heal. (2014) 55:705–12. 10.1016/j.jadohealth.2014.04.016 24925491 PMC4380744

[30] Patton GC Johnson-Sabine E Wood K. Abnormal eating attitudes in London schoolgirls–a prospective epidemiological study: outcome at twelve month follow-up. Psychol Med. (1990) 20:383–94. 10.1017/S0033291700017700 2356264

[31] Unsain RF Sato P de M Ulian MD Sabatini F Oliveira MS Scagliusi FB. Triangulation of qualitative and quantitative approaches for the study of gay bears' food intake in São Paulo, Brazil. Qual Res J. (2021) 21:444–55. 10.1108/QRJ-04-2020-0034

[32] Hidayah GN Syahrul Bariah AH. Eating attitude, body image, body composition and dieting behaviour among dancers. Asian J Clin Nutr. (2011) 3:92–102. 10.3923/ajcn.2011.92.102

[33] Kulshreshtha M Babu N Goel NJ Chandel S. Disordered eating attitudes and body shape concerns among North Indian Kathak dancers. Int J Eat Disord. (2021) 54:148–54. 10.1002/eat.23425 33283330

[34] Caminada E. History of Dance: Cultural Revolution. 1st ed. Rio de Janeiro: Sprint (1999).

[35] Dantas AG Alonso DA Sánchez-Miguel PA del Río Sánchez C. Factors dancers associate with their body dissatisfaction. Body Image. (2018) 25:40–7. 10.1016/j.bodyim.2018.02.003 29475190

[36] Alexias G Dimitropoulou E. The body as a tool: professional classical ballet dancers' embodiment. Res Dance Educ. (2011) 12:87–104. 10.1080/14647893.2011.575221

[37] Ferguson CJ Muñoz ME Garza A Galindo M. Concurrent and prospective analyses of peer, television and social media influences on body dissatisfaction, eating disorder symptoms and life satisfaction in adolescent girls. J Youth Adolesc. (2014) 43:1–14. 10.1007/s10964-012-9898-9 23344652

