# Supplementary material for: “Can A Ballerina Eat Ice Cream?”: A Mixed-Method Study on Eating Attitudes and Body Image in Female Ballet Dancers
Source: Front Nutr. 2022 Jan 6;8:665654. doi: 10.3389/fnut.2021.665654 (PMC8770265; doi:10.3389/fnut.2021.665654)
Supplement: Supplementary file 1 [file Data_Sheet_1.pdf]

Focus Groups Script

Hello everyone, everything OK? Well, you already filled out some questionnaires and now we will talk about various issues related to their content. It is important that you keep in mind that there are no ‘right’ or ‘wrong’ answers to what I am going to ask, your answers and opinions are important to me and I am very interested in getting to know you and hear you all.

How many hours do you practice each day? How many times a week? What do you think of your load of rehearsals? [psychological and physical stress; expected results] **What do your family and friends think about this?** [supported or not; if not, how do you deal with it] **If you could, would you change something in your routine?**

How do you usually eat on a daily basis? [where they feed; has a cafeteria; if so, who proposes food; what can you eat and what can't you eat? - RULES - who imposes] **Does this change according to the time of rehearsals (pre- or post-competition, presentation, vacation)?** [what changes; if so, who proposes the change - they, athletes, coach, nutritionist] **Is this always the case? How do you feel and what do you think about it?** [causes some discomfort; like it] **Have you tried to do some kind of diet? And if so, how was it and what motivated you to do it?** [reasons; had encouragement]

In some moments, although we are not hungry, we feel “desire to eat” certain foods. How do you deal with this feeling? Does this sensation suddenly appear, or are you able to connect it to any situation (s)? Do you feel this "desire" for things other than food? [what; how they deal with it]

What is a pleasant meal for you? [pleasant foods - why; companies - why; environments - why] **When it comes to foods considered pleasurable, what do you feel when you eat them?** [have a problem; fault; fear of getting fat - why] **How do people (family, friends, coach, other athletes) behave when you eat these foods?** [external pressures]

Do you see any differences between your usual diet and non-dancers’ diets? Other people usually make comments about what you eat?

Think about the most important competition or presentation you had this year. Do you remember what you ate right after or the day after the competition ended?

How do you feel about your body? [how they perceive the body; like it or not - why] **Do you always feel that way?** [speculate in relation to pre- and post-competition or vacation periods; in ballet; day to day]

How do you perceive your body to practice classical ballet? [ideal; bad; would change something - what] **Do you have any goals or inspiration? And if so, what motivates you to want this?**

Do you have goals for your career? What would these objectives be? And what do you need to do to get them? [check if issues related to food and the body arise]

From what you have just said you have a very ruled lifestyle**. Thinking about stopping dancing, one day, would you keep the same routine (training and food)? Even if that choice has an impact on the body, would you maintain the same answer?**

Would anyone like to add anything else? Well, that is it. I would like to thank you for your presence and for sharing with me all this valuable information about you all.
